# Spatial Competence and Brain Plasticity in Congenital Blindness via Sensory Substitution Devices

**DOI:** 10.3389/fnins.2020.00815

**Published:** 2020-07-30

**Authors:** Daniel-Robert Chebat, Fabien C. Schneider, Maurice Ptito

**Affiliations:** ^1^Visual and Cognitive Neuroscience Laboratory (VCN Lab), Department of Psychology, Faculty of Social Sciences and Humanities, Ariel University, Ariel, Israel; ^2^Navigation and Accessibility Research Center of Ariel University (NARCA), Ariel, Israel; ^3^Department of Radiology, University of Lyon, Saint-Etienne, France; ^4^Neuroradiology Unit, University Hospital of Saint-Etienne, Saint-Etienne, France; ^5^BRAIN Lab, Department of Neuroscience and Pharmacology, University of Copenhagen, Copenhagen, Denmark; ^6^Chaire de Recherche Harland Sanders en Sciences de la Vision, École d’Optométrie, Université de Montréal, Montréal, QC, Canada

**Keywords:** multisensory, spatial cognition, vision, touch (haptic/cutaneous/tactile/kinesthesia), sensory substitution, brain plasticity, congenital blindness, navigation

## Abstract

In congenital blindness (CB), tactile, and auditory information can be reinterpreted by the brain to compensate for visual information through mechanisms of brain plasticity triggered by training. Visual deprivation does not cause a cognitive spatial deficit since blind people are able to acquire spatial knowledge about the environment. However, this spatial competence takes longer to achieve but is eventually reached through training-induced plasticity. Congenitally blind individuals can further improve their spatial skills with the extensive use of sensory substitution devices (SSDs), either visual-to-tactile or visual-to-auditory. Using a combination of functional and anatomical neuroimaging techniques, our recent work has demonstrated the impact of spatial training with both visual to tactile and visual to auditory SSDs on brain plasticity, cortical processing, and the achievement of certain forms of spatial competence. The comparison of performances between CB and sighted people using several different sensory substitution devices in perceptual and sensory-motor tasks uncovered the striking ability of the brain to rewire itself during perceptual learning and to interpret novel sensory information even during adulthood. We discuss here the implications of these findings for helping blind people in navigation tasks and to increase their accessibility to both real and virtual environments.

## Introduction

Several different mechanisms influence the development of the congenitally blind brain. Neuroimaging techniques show that brain structures devoted to vision are greatly affected (Kupers and Ptito, 2014; Fine and Park, 2018; Singh et al., 2018), and that the extensive use of the remaining senses (e.g., touch or/and audition) helps blind people to develop a set of impressive skills in various cognitive tasks, probably due to the triggering of neural plasticity mechanisms (Schinazi et al., 2016). These enhanced behavioral performances are correlated to brain plasticity using various types of SSDs (Chebat et al., 2018a). Brain modifications are triggered by sensory deprivation and later by the training of the other senses, for example through the use of SSDs to “perceive” visual information. We perceive our environment using all of our senses in parallel, creating a rich multisensory representation of space (Chebat, 2020), but how does the complete lack of vision impact spatial competence and spatial learning? In this paper, we review the plastic changes that occur in the brain of CB that are triggered by SSDs use.

## Sensory Substitution Devices (SSDs)

**FIGURE 1:**
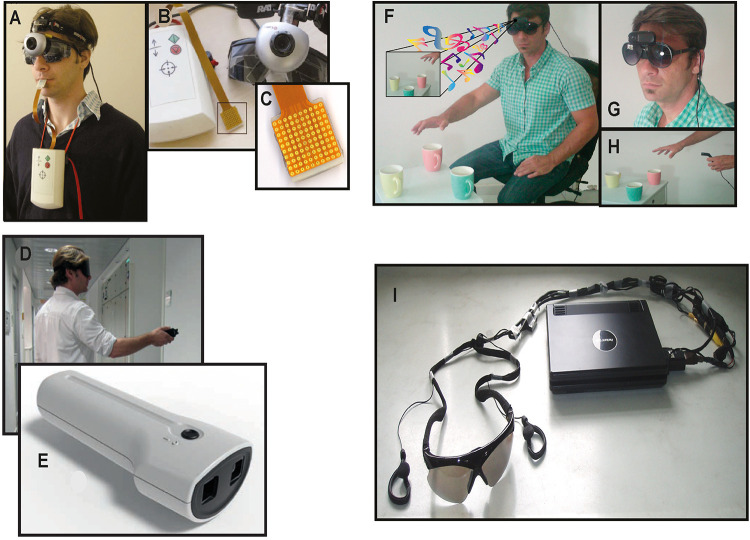
Sensory Substitution Devices (SSDs). Examples of the experimental setup for several different sensory substitution devices. **(A–C)** The Tongue Display Unit (TDU). **(A)** The camera mounted on a pair of blindfold-glasses. **(B)** The entire setup with camera, image converter box, and tongue grid. The box, which is worn on the chest, controls the intensity of the electrotactile stimulation. **(C)** The tongue grid. Applied to the tongue, it delivers a tingling sensation through the electrodes. **(D)** A participant holding the EyeCane that delivers vibrations and sounds to indicate the distance to an object. **(E)** The sensors of the EyeCane and device. **(F)** The EyeMusic experimental setup with headphones and camera. **(G)** The head mounted camera of the EyeMusic. **(H)** The EyeMusic converts colors into different sounds, enabling the recognition of the red apple among the green ones. **(I)** vOICe apparatus. Converts visual images into soundscapes (Meijer, 1992).

SSDs translate visual cues into tactile or auditory information. SSDs consist of three components: a sensor, a processing unit that converts the visual cues using a specific code and algorithm, and a delivery system to transmit the tactile or auditory information. SSDs differ in terms of their respective approaches, codes or algorithms for capturing and sending information, and also in terms of their specific components, but they all aim to transmit visual information via another sense. For example, SSDs use different kinds of sensors to capture visual information, either from a camera (Bach-y-Rita et al., 1969; Meijer, 1992; Bach-y-Rita and Kercel, 2003; Ptito, 2005; Chebat et al., 2007a; Mann et al., 2011; Figures 1A,F,I for images of the camera set-ups used for the TDU, EyeMusic, and vOICe) or sonic (Kay, 1974), ultrasonic (Shoval et al., 1998; Hill and Black, 2003; Bhatlawande et al., 2012) and infrared sensors (Dunai et al., 2013; Maidenbaum et al., 2014c; Stoll et al., 2015). The means to deliver the information to the user can also vary greatly. In the case of the Tongue Display Unit (TDU) (Bach-y-Rita et al., 1969; Bach-y-Rita and Kercel, 2003; Figures 1A–C), the image captured by a camera is translated and coded onto an electro-tactile grid which “draws” an image on the tongue of the user (Figure 1C). In the case of the EyeCane (Maidenbaum et al., 2014c), distance information is received from an infra-red sensor and delivered to the hand and ears through the frequency of vibrations or sounds (Figures 1D,E). The EyeMusic (Abboud et al., 2014; Figures 1F–H) and vOICe (Meijer, 1992; Figure 1I) also rely on a camera for visual information but the algorithm codes the images into sounds, and in the case of the EyeMusic, different musical instruments code for different colors in the image.

Despite these differences, SSDs all use a form of code to translate visual information that must be actively integrated by the user. This process, called distal attribution (Auvray et al., 2005) requires the reinterpretation of what seems like random stimulation into a coherent, visual percept through sensori-motor feedback (Chebat et al., 2018a). This form of reinterpretation of visual information has often been likened to a kind of learned synesthesia (Ward and Wright, 2014). The use of these devices to transfer visual information, via the tactile, auditory or vibratory channels, coupled with complete congenital sensory deprivation leads to training-induced recruitment of brain regions that were typically considered purely visual (Ptito, 2005; Amedi et al., 2007; Proulx et al., 2016). Although the phenomenological sensations reported by CB during the use of these devices is similar to vision (Chebat et al., 2018a), these devices cannot approximate the complexity and resolution of vision *per se*. Thus, the resulting sensations are very different from vision in the sighted, and cannot genuinely replace a missing sense for all of its functions (Moraru and Boiangiu, 2016). This is also true for task specific sensory independent regions according to the task being completed (Kupers et al., 2010a; Matteau et al., 2010; Ptito et al., 2012; Striem-Amit et al., 2012a, b; Abboud et al., 2015; Maidenbaum et al., 2018). SSDs have not become widespread in their general use by the blind population (Loomis et al., 2010; Elli et al., 2014), for various practical reasons (Chebat et al., 2018a). In order for an SSD to be widely accepted by the a visually impaired public, it needs to meet many several criteria, such as general use (for many tasks), facility of use, cost and be worth the learning process in terms of the visual information it can afford in real time (Chebat et al., 2018a). From the point of view of navigation, several of these devices have great potential in improving navigation competence and strategies used by blind people during navigation. We review these concepts in the following sections.

## Sensory Deprivation, Brain Plasticity, Amodality and Spatial Cognition

**FIGURE 2:**
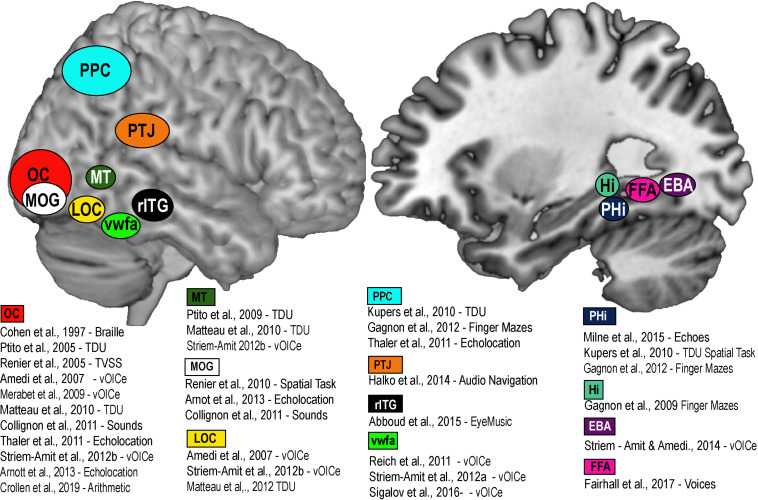
Brain Amodality and Task specificity via SSDs. A schematic representation of task specific sensory independent recruitment of brain areas via SSDs, or other codes (Braille, echolocation, etc.). Placement of brain areas are approximative. PPC, Posterior parietal cortex; OC, Occipital Cortex; MOG, Medial Occipital Gyrus; LOC, Lateral Occipital Gyrus; MT, Medial Temporal; VWFA, Visual Word Form Area; rITG, Right Infero-Temporal Gyrus; PTJ, Parietal Temporal Junction; PHi, Parahippocampus; Hi, Hippocampus; FFA, Fusiform Face area; EBA, Extrastriate Body Area.

A large part of the cortical mantle is dedicated to vision. In the macaque, about 55% of the entire cortex is in some way responsive to visual information, and in humans it is about 35%. This cortical space is by no means wasted for people who are blind from birth, and can be recruited in a variety of cognitive and spatial tasks using the remaining intact senses. Indeed, the recruitment of primary visual areas by other sensory modalities has been known for quite some time in CB (Kupers and Ptito, 2014). This process, known as *amodality* (Heimler et al., 2015; Chebat et al., 2018b) enables the recruitment of brain areas in a task specific, sensory independent fashion (Cohen et al., 1997). The recruitment of task-specific brain nodes for shapes (Ptito et al., 2012), motion (Saenz et al., 2008; Ptito et al., 2009; Matteau et al., 2010; Striem-Amit et al., 2012b), number-forms (Abboud et al., 2015), body shapes (Striem-Amit and Amedi, 2014), colors (Steven et al., 2006), word shapes (Striem-Amit et al., 2012a), faces (Likova et al., 2019), echolocation (Norman and Thaler, 2019), and tactile navigation (Kupers et al., 2010a; Maidenbaum et al., 2018) is thought to represent mechanisms of brain plasticity (Fine and Park, 2018; Singh et al., 2018) for specific amodal recruitment (Ptito et al., 2008a; Chebat et al., 2018b; see Figure 2). The recruitment of the brain areas via SSDs not only shows that it is possible to supplement missing visual information, but that the brain treats the SSD information as if it were real vision, in the sense that it tries to extract the relevant sensory information for each specific task we are trying to accomplish (i.e., motion, colors, navigation, and other tasks illustrated in Figure 2). How do brain plasticity and amodality influence spatial perception in people who are blind from birth? Since, vision is quite important for active navigation (McVea and Pearson, 2009; Ekstrom, 2015; Jeamwatthanachai et al., 2019), how essential is it for the development of spatial abilities and the neural networks that support these abilities?

**FIGURE 3:**
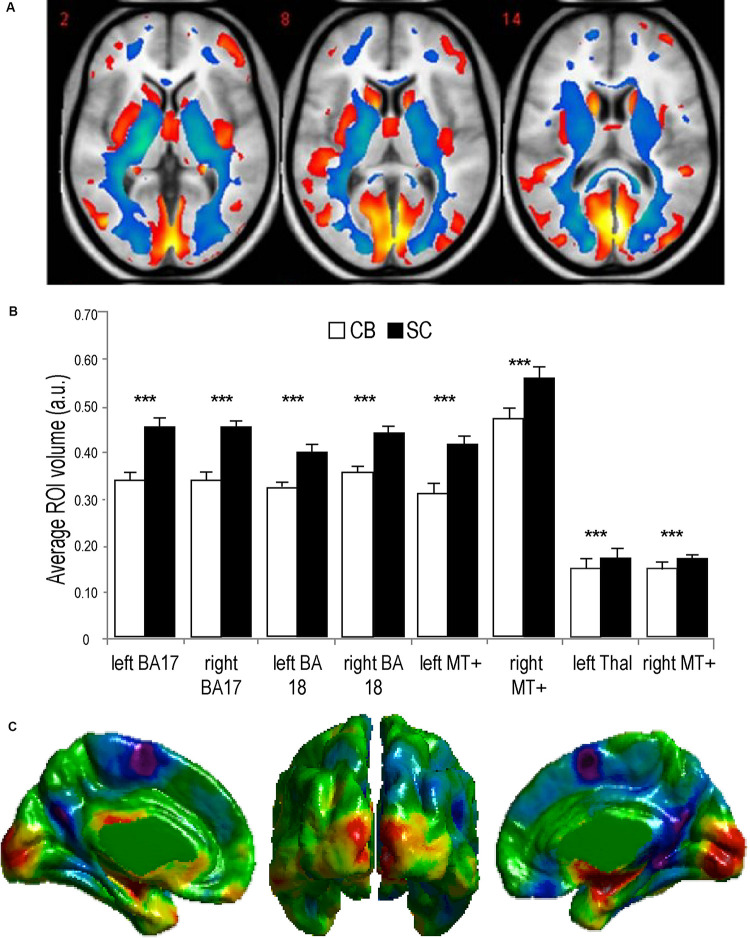
Anatomy of the visual system in congenital Blindness. **(A)** Voxel-based Morphometry results illustrate reductions in white matter projections (blue) and visual cortices (red). **(B)** Bar charts summarize the volumetric reductions in various visual cortical regions for congenitally blind (CB) and sighted controls (SC) (adapted from Ptito et al., 2008b). **(C)** Cortical thickness measurements indicate a thicker visual cortex in CB (adapted from Kupers and Ptito, 2014). ***p < 0.001.

Animals can use either visual, tactile (Pereira et al., 2007), olfactory (Save et al., 2000), vestibular (Etienne and Jeffery, 2004), or auditory (Ulanovsky and Moss, 2008) cues to navigate (Rauschecker, 1995). Indeed, prolonged visual impairment improves auditory spatial acuity in ferrets (King and Parsons, 2008). Humans on the other hand have mostly relied on the visual sense to navigate, and vision is considered as the most adapted spatio-cognitive sensory modality (Foulke, 1982). Vision is a capital tool to form cognitive maps (Strelow, 1985). The more these cues are salient in terms of color, or shape the easier they are remembered, and the more precise is our representation of the environment (Appleyard, 1970). Vision is thus helpful for spatial representations, and also for obstacle avoidance. When approaching an obstacle, visual cues guide foot placement by constantly updating our distance with the obstacle (Patla, 1998; Patla and Greig, 2006) and adapt our locomotive behavior according to the circumstance (Armand et al., 1998; MacLellan and Patla, 2006). Certain auditory and tactile spatial abilities are also compromised by the lack of visual experience (Zwiers et al., 2001; Gori et al., 2014). For example, CB individuals show auditory and proprioceptive spatial impairments (Cappagli et al., 2017), deficits in auditory spatial localizations (Gori et al., 2014), and in encoding spatial motion (Finocchietti et al., 2015). It is the lack of visual information that leads to differences in the normal development and alignment of cortical and subcortical spatial maps (King and Carlile, 1993; King, 2009) and appropriate integration of the input from the remaining sensory modalities (Cattaneo et al., 2008; Gori et al., 2014). In addition, most of the neuronal networks responsible for spatial tasks are volumetrically reduced (Figure 3; Noppeney, 2007; Ptito et al., 2008b) compared to the sighted, including the posterior portion of the hippocampus (Chebat et al., 2007a; Illustrated in Figure 6A), which suggests that the taxing demands of learning to navigate without vision drives hippocampal plasticity and volumetric changes in CB (Chebat et al., 2007a; Ptito et al., 2008a; Leporé et al., 2010). Furthermore, there is a cascade of modifications involving other non-visual brain structures that undergo anatomical (Yang et al., 2014), morphological (Park et al., 2009), morphometric (Rombaux et al., 2010; Tomaiuolo et al., 2014; Aguirre et al., 2016; Maller et al., 2016), and functional connectivity (Heine et al., 2015) alterations.

**FIGURE 4:**
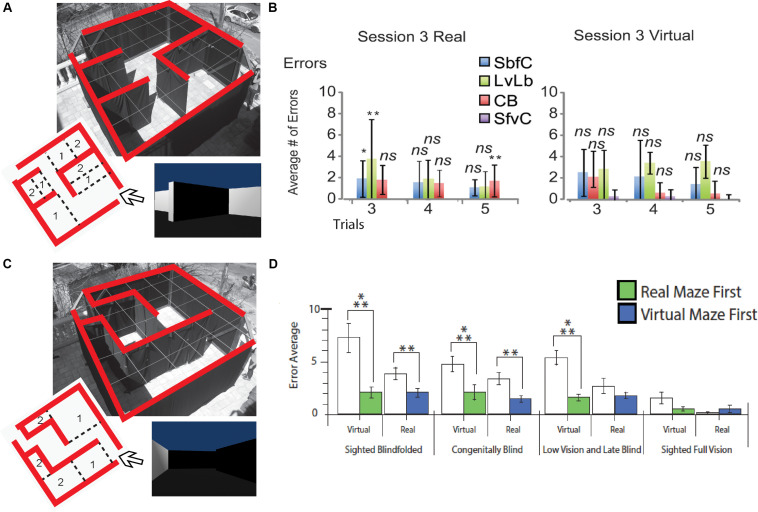
Behavioral Studies. Schematic representation of behavioral studies using the EyeCane. **(A)** A Hebb Williams maze configuration used to test participants’ ability to learn a configuration over several days of training in real and virtual environments. **(B)** Behavioral results showing performance for CB, sighted blindfolded controld (SbfC), low vision and late blind participants (LvLb), and sighted full vision controls (SfvC). On the third day of training, there is mostly a lack of statistical difference with the performance of the sighted using vision. **(C)** A Hebb Williams maze configuration used for testing the transfer of spatial knowledge between real and virtual environments and vice versa. **(D)** Behavioral results showing the transfer of spatial knowledge between real and virtual environments and vice versa for CB, and LB (adapted from Chebat et al., 2015, 2017). *p < 0.05 and **p < 0.01.

Despite these anatomical changes, visual experience is not necessary for the development of topographically organized maps of the face in the intraparietal cortex (Pasqualotto et al., 2018), or for the ability to represent the work space (Nelson et al., 2018). CB can form mental representations of the work space via haptic information as efficiently as sighted people, indicating that this ability does not depend on visual experience (Nelson et al., 2018). People who are congenitally blind are capable of avoiding obstacles (Kellogg, 1962; Chebat et al., 2011, 2020), integrating paths (Loomis et al., 2012), remembering locations (Chebat et al., 2015), and generating cognitive representations of space (Passini et al., 1990; Thinus-Blanc and Gaunet, 1997; Fortin et al., 2006; Chebat et al., 2018a, b). As a consequence, CB maintain the ability to recognize a familiar route and represent spatial information (Marmor and Zaback, 1976; Kerr, 1983; Passini et al., 1990; Loomis et al., 1993; Thinus-Blanc and Gaunet, 1997; Fortin et al., 2006; Leporé et al., 2009). Moreover, CB can even perform better than their blindfolded sighted counterparts in certain spatial tasks (Rieser et al., 1980; Passini et al., 1990; Loomis et al., 1993; Thinus-Blanc and Gaunet, 1997) and navigate by substituting vision with echolocation (Supa et al., 1944; Teng et al., 2012; Kolarik et al., 2017), tactile information (White et al., 1970; Kupers et al., 2010a; Chebat et al., 2011, 2015, 2017), or even proprioceptive information (Juurmaa and Suonio, 1975). Interestingly, neonatal visual deprivation does not impair the cognitive representation of space. Instead, when substituting visual information by the tactile or auditory modality via SSDs, similar performances are observed in CB compared to sighted participants (Chebat et al., 2018a). CB are therefore able to navigate efficiently using either audition (Maidenbaum et al., 2014b, c, d; Chebat et al., 2015; Bell et al., 2019) or touch (Chebat et al., 2007a, 2011, 2020; Kupers et al., 2010b). They can locate objects (Auvray and Myin, 2009; Chebat et al., 2011), navigate around them (Chebat et al., 2011), and even perform as well (Chebat et al., 2015, 2017) or better than the sighted in certain spatial tasks (Loomis et al., 1993; Chebat et al., 2007b, 2015, 2017). These abilities can be further improved with training (Likova and Cacciamani, 2018). For instance, spatial knowledge can be acquired by CB individuals by using sound cues while playing video games and transferred to the real world (Connors et al., 2014). Using the EyeCane (Figures 1D,E), congenitally blind participants can learn real and virtual Hebb-Williams mazes as well as their sighted counterparts using vision (Chebat et al., 2015; Figures 4A,B). When learning an environment in the virtual world CB participants are able to create a mental map of this environment which enables them to resolve the maze in the real world more efficiently, and vice versa. Moreover, they can transfer the acquired spatial knowledge from real to virtual mazes (and conversely) in the same manner as the sighted (Figures 4C,D; Chebat et al., 2017).

Taken together, these results indicate that even if certain specific spatial abilities are deficient in the case of congenital blindness, the resulting deficit in navigation still remains purely perceptual (Vecchi et al., 2004; Amedi et al., 2005), and not as previously suggested a cognitive deficit (von Senden, 1932).

## Navigation: Strategies for Acquiring Spatial Knowledge

Navigation is the ability to find our way in the environment (Sholl, 1996; Maguire et al., 1999) and requires several distinct, yet interrelated skills. Navigation is associated with different perceptual, cognitive and motor networks for path integration, wayfinding or obstacle avoidance and detection. For navigation through the environment, animals and humans alike must translate spatial information into cognitive maps that they compare with an internal egocentric representation (Whitlock et al., 2008). Animals can use strategies to navigate using olfactory indices (Holland et al., 2009), more complex egocentric strategies like the integration of paths based on proprioceptive cues (Etienne and Jeffery, 2004), or strategies relying on complex cognitive maps based on the spatial relation that objects have with one another (O’keefe and Nadel, 1978). Allocentric frames of reference are an abstract coordinate system enabling one to navigate from point to point, whereas an egocentric one does not (Klatzky, 1998).

Several types of labyrinths and mazes (Hebb and Williams, 1946; Barnes et al., 1966; Morris, 1984) and many other variants, including virtual mazes (Shore et al., 2001) have been used to understand the process by which people resolve spatial problems. The Morris maze has particularly been used (Cornwell et al., 2008), often to test the navigational ability of human subjects and its neurological substrates (*see: section on neurological substrates of navigation*). There is, however, a large inter-subjects variability in navigational performances (Wolbers and Hegarty, 2010), that can be attributed to the type and variety of strategies used when navigating. A navigational strategy is defined as a set of functional laws used in order to reach a spatial goal. It influences the way we interact with the environment and our representation of space. In other words, cognitive maps are largely dependent on the employed navigational strategies. Experienced navigators are usually better (Hegarty et al., 2006) since they employ more diverse strategies (Kato and Takeuchi, 2003; Blajenkova et al., 2005), and they are more flexible concerning the strategy to be adopted (Saucier et al., 2003). O’keefe and Nadel (1978) identified different strategies in the behavior of rats while exploring the environment in a Morris water maze. These strategies include the exploration of a novel environment as well as the detection of changes in an already familiar environment, and the ability to make detours or create shortcuts. In sighted humans, three major orientation strategies have been identified using the same paradigm (Kallai et al., 2005). They are characterized by a set of behaviors while looking for a platform in an open space. 1. *Thigmotaxis* (following the wall and approaching the platform); 2. *Turning in circles* (wandering around in circles); 3. *Visual scans* (turning in place to change their view-point); 4. *Enfilade* (accomplishing a quick scan and moving directly to the platform).

In blindness, research on orientation and mobility have identified a series of strategies used in navigation and the exploration of non-familiar environments reminiscent of what has been reported in sighted people (Geruschat and Smith, 1997). Hill et al. (1993) asked blind and low vision participants to explore an open space, find four objects and remember their emplacement. The movement of participants was recorded, quantified and categorized into different strategies. Certain of these strategies apply specifically to people with low vision, and others to blind individuals. Strategies were assigned to five categories for blind participants (Schinazi et al., 2016). 1. *Perimetry* (searching for objects by moving alongside the walls, or the perimeter of the room); 2. *Perimetry toward the center* (moving in concentric circles from the periphery toward the center of the room); 3. *Grid* (exploring the space in a systematic grid-like fashion); 4. *Cyclical* (moving directly from one object to the next); 5. *Perimetry to the object* (moving from the periphery toward the object).

The differences in strategies employed by sighted and blind people reflect the restrictions imposed on navigation without sight; there is no fundamental difference between the strategies employed by the blind and sighted, the only notable difference is that blind people cannot perform visual scans to find their targets, they must rely on encoding of stimuli using egocentric rather than allocentric, coordinates (Röder et al., 2008; Pasqualotto and Proulx, 2012). Although these strategies encourage an egocentric representation of space, and visual experience facilitates allocentric representations (Pasqualotto et al., 2013), it is also possible to achieve an allocentric representation of space without vision. The last two strategies, *cyclical* and *perimetry to the object*, that require an allocentric representation, can only be used by blind people once they have become familiar with the environment using the other strategies.

## Neural Correlates of Navigation

Sighted people often accomplish tasks of navigation with the greatest ease, like for example going to a well-known destination, or to avoid obstacles in a crowded hallway. This seemingly effortless behavior is in fact the result of the interaction of a complex network of brain regions integrating information from visual, proprioceptive, tactile and auditory sources which translate into the appropriate behavior (Tosoni et al., 2008). The brain takes into consideration information from various senses simultaneously and accomplishes a multitude of operations to enable someone to find their way or step over an obstacle. The hippocampal and parietal cortices are two regions that are traditionally viewed as being related to spatial tasks (Poucet et al., 2003) since they are involved in the processing (Rodriguez, 2010) and in the encoding (Whitlock et al., 2008) of high level spatio-cognitive information, which is crucial for navigation.

### The Hippocampus

The hippocampus is part of the medial temporal lobe and is implicated in spatial memory. In the adult monkey, a lesion to the hippocampus results in a deficiency in spatial learning (Lavenex et al., 2006), and in humans, its enlargement predicts learning of a cognitive map (Schinazi et al., 2013), which confirms its functional role in navigation. When implanting electrodes into the medial temporal lobe of rats that can freely move in a maze, pyramidal cells in the hippocampus respond preferentially when the animal is in a precise place (O’Keefe and Dostrovsky, 1971). These place cells, which are mostly found in the posterior part of the hippocampus (O’Keefe and Speakman, 1987; Burgess and O’Keefe, 1996), are organized in functional units that represent space (O’keefe and Nadel, 1978). They are at the origin of cognitive maps of the environment. Space is cartographied using a matrix of pyramidal cells that respond preferentially to places having been already visited (O’Keefe and Burgess, 2005). These maps are allocentric (O’Keefe, 1991) and use the limits of traversable space of their environment (O’Keefe and Burgess, 2005). These cells are also found in the primate (Matsumura et al., 1999) and can represent the position of objects and landmarks of the environment (Rolls and Kesner, 2006). These place cells can also adjust their response according to changes in the environment (Lenck-Santini et al., 2005) and the position of objects in a labyrinth (Smith and Mizumori, 2006). In addition, the prefrontal cortex (PFC) also seems to be sensitive to places, like hippocampal cells (O’Keefe and Dostrovsky, 1971).

In addition to place cells, there also exists populations of cells that are coding for the heading direction (Taube et al., 1990; Oler et al., 2008). Path integration requires that the animal constantly updates its direction during its movements through its trajectory. These cells that code for the direction of an animal are found in the subiculum (Taube et al., 1990), in the striatum (Wiener, 1993) and in the posterior parietal cortex (Chen et al., 1994). These cells compose a sort of internal compass that allows the animal to monitor its direction while traveling.

### The Parahippocampal Complex

The human parahippocampus is composed of the entorhinal and perirhinal cortex. This structure surrounds the hippocampus, and the entorhinal cortex is one of the important sources of projection to the hippocampus. It is also implicated in navigation (Aguirre et al., 1996). The entorhinal cortex is composed of Brodmann area 28 and is situated alongside the rhinal sulcus. The grid cells (Hafting et al., 2005) recorded in the dorsal part of the entorhinal cortex respond preferentially in an organized way and code the environment in the form of a grid. They have receptive fields that are sensitive to different parts of the environment, which are divided in quadrants, like a grid. In opposition to place-cells of the hippocampus, the entorhinal grid-cells code the environment in a geometric fashion (Moser et al., 2008). The hippocampus and the entorhinal cortex cooperate to allow for navigation and we know that this system, when lesioned, perturbs this function (Parron et al., 2006). Indeed, sighted human patients with lesions to the parahippocampus are incapable of learning a new route (Hublet and Demeurisse, 1992; Maguire, 2001). In fact, a case study demonstrates that a lesion to the hippocampus has an effect mostly on the allocentric representation of a path (Holdstock et al., 2000). The parahippocampal area is also involved in the recognition of visual scenes used to navigate (Epstein and Kanwisher, 1998; Epstein et al., 2007). By representing an image of visual scenes to participants in an fMRI scanner, there is an elevation of blood flow in the parahippocampus, leading to the coining of this region as the parahippocampal place area (PPA).

It was later discovered that cells that are sensitive to places are also found in the retrosplenial cortex (RS) (Epstein, 2008). Although RS and PPA are both sensitive to the recognition of visual scenes for navigation, they have complementary, yet different roles (Epstein et al., 2007). The PPA would be more involved in the recognition of scenes, namely the representation of a particular one during navigation, whereas, the retrosplenial cortex serves to situate that scene in the environment. This type of scene recognition is used during navigation to transmit information (an egocentric representation) to a representation of this place on a map (allocentric). The interaction of these two zones during navigation could therefore serve to transform egocentric information of the environment into an allocentric one (Epstein, 2008). These landmarks that are so important for the formation of cognitive maps are coded in the parahippocampus in order to be recognized in their context and by the retrosplenial cortex to be situated in space.

### The Parietal Cortex

The parietal cortex allows for several different functions. The anterior part of the parietal cortex is responsible for the integration of somatosensory information (Tommerdahl et al., 2010), and the posterior part (PPC) is implicated in multimodal integration of spatial information (Cohen, 2009), that is used to explore personal space (Mountcastle et al., 1975). PPC is also involved in spatial navigation (Seemungal et al., 2008). Lesion studies in the parietal cortex in rodents (King and Corwin, 1993) and primates (Weniger et al., 2009) demonstrate deficits in the processing of egocentric information: animals cannot integrate a path (Save et al., 2001). The PPC is part of the dorsal visual stream (Mishkin et al., 1983), and enables the perception of movement and the planification of our own movement (Goodale and Milner, 1992). The transformation of our own allocentric representation into a representation centered on the self to plan our movement in space takes place in the PPC (Buneo and Andersen, 2006). In monkeys, neural activity in the parietal cortex is sensitive to the direction of a learned trajectory (Crowe et al., 2004a), and these cells are activated when the animal tries to solve a maze (Crowe et al., 2004b). A recent model on the role of the parietal cortex suggests that it would interact with the hippocampus to select a more appropriate route between two points (planification), and produces a representation that is egocentric of the environment to guide movement between those two points (execution) (Nitz, 2009). Moreover, the parietal cortex interacts with the frontal cortex for the planification and decision making).

Clinical studies also show the importance of the parietal cortex in navigation and spatial representation in general (De Renzi, 1982a, b). Lesions in parietal regions in humans can lead to spatial disorientation (Hublet and Demeurisse, 1992), meaning an inability to find one’s way in the environment, and in some occasions even spatial (Vallar and Calzolari, 2018) or personal neglect (Committeri et al., 2018). fMRI studies showed that the parietal cortex is activated multiple times during the navigation process (Spiers and Maguire, 2006). Medio-Parietal regions play an important role in analyzing movement in immediate space and parietal regions play a role in the opacification of movement in space that is not visually accessible (Spiers and Maguire, 2006). This explains why lesions in the parietal lobe interfere with movement in personal space (spatial neglect) and in navigational space (topographical disorientation) as well. Studies using tactile mazes found that the parietal cortex is essential for the acquisition of spatial memory and the planification of movement (Saito and Watanabe, 2006). Indeed, in this task, participants use their parietal cortex only in the encoding of the goal phase of the task, meaning the encoding of the exit and the planification of movement to reach it.

## Neural Activity According to the Type of Navigation Strategy

Using fMRI, the hippocampus in humans has been shown to be implicated in navigation (Ghaem et al., 1997). When participants try to solve a maze while in the scanner, the recorded activity is stronger in the right hippocampus (Maguire et al., 1997; Gagnon et al., 2012). Many studies have involved the hippocampus in topographic memory of places (Burgess et al., 2002) and allocentric representations (O’Keefe, 1991; Holdstock et al., 2000). A study demonstrated that the modulation of the interaction between the hippocampus and frontal or parietal regions depends on the type of strategy used in navigation (Mellet et al., 2000). Indeed, it is confirmed that the cortical activity in navigation tasks depends on the ability and strategies used by participants (Ohnishi et al., 2006). In addition, the cerebellum has also been linked to navigational tasks (Rondi-Reig et al., 2014).

There are also differences between men and women according to the strategy used to navigate (Grön et al., 2000). Men and women do not employ the same strategies when navigating, and men perform in general better than women (Astur et al., 1998). These differences are attributable to the fact that men employ strategies that are mostly allocentric and that women use more egocentric strategies to navigate (Sandstrom et al., 1998). BOLD responses differ when the mental navigation of maps are allocentric or from an egocentric viewpoint of a route (Mellet et al., 2000). Indeed, positron emission tomography (PET) shows that the hippocampus on the right side and the fronto-parietal network are recruited for both egocentric and allocentric representations (Galati et al., 2000; Zaehle et al., 2007). The PPA is activated bilaterally only for egocentric tasks. Using fMRI, different activations for egocentric and allocentric navigations are also found, but with certain nuances (Shelton and Gabrieli, 2004). In a fMRI study, Holdstock et al. (2000) reported that the hippocampus is more activated by allocentric tasks, and confirmed previous data reported in humans and animals (O’Keefe, 1991). In this study, the authors show that a parietal network is involved in navigation in both conditions, but that the frontal region is only present in the egocentric condition. It was found that participants that performed well in spatial tasks use allocentric strategies that are positively correlated with the medial temporal lobe (hippocampus). In opposition participants that performed poorly activated the parietal cortex and used more egocentric strategies (Ohnishi et al., 2006).

## The Impact of Visual Deprivation on Spatial Competence: The Case for the Convergent Model

**FIGURE 5:**
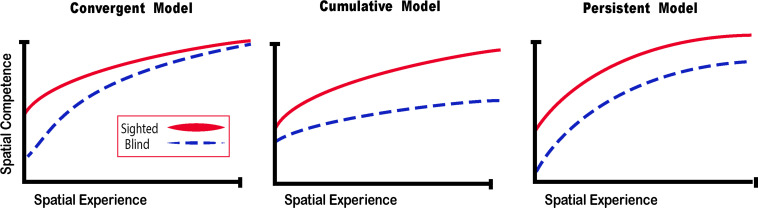
Spatial Competence Acquisition Models for the Blind. The convergent model holds that spatial competence of CB in novel environments eventually reaches the level of the sighted with enough experience. The cumulative model considers that errors made by blind people when exploring space are cumulative, therefore even by acquiring more spatial experience, their spatial competence can never equal that of the sighted. The persistent model projects that errors made by the blind during spatial explorations are persistent and that their spatial competence remains below that of the sighted. In this review, we argue for the convergent model for the acquisition of spatial competence by the blind (adapted from Schinazi et al., 2016).

What happens then when someone is deprived of vision since birth? It is more difficult to gather sensory information in the absence of vision, and that information is harder to interpret, but spatial representations and competence can still be achieved. If sensory information is substituted with a different modality, the convergent model (Schinazi et al., 2016) suggests that spatial competence can be acquired faster (Figure 5).

### Theories on the Acquisition of Spatial Competence in Blindness

Interestingly enough, as early as 1779, Diderot noted in his *letter on the blind*, the ability of certain non-sighted people to orient themselves in space without the aid of a cane, and that they had a certain innate sense for the perception of obstacles. In 1944, studies at Cornell University (Supa et al., 1944) showed that blind people were capable of detecting obstacles only when they were provided auditory information. The absence of tactile information did not perturb their obstacle detection sense, but the absence of any auditory information was detrimental to their performance. This hypothesis was confirmed by Ammons et al. (1953) who showed that in blind people in whom the auditory input was blocked, there was an inability to perceive obstacles. They concluded that audition was a crucial factor for navigation in blindness. This phenomenon is called echolocation. Blind people still use this technique by tapping their cane on the ground, clapping their hands or making clicking sounds with their tongue to perceive echoes. Kellogg (1962) was the first to quantify this ability. He measured the sensitivity of blind and sighted volunteers to the variation of size, distance and texture of objects perceived only with auditory echoes. He demonstrated that blind people had significantly superior results compared to the sighted in terms of their ability to detect objects, their texture and distance (Kellogg, 1962). These results were reproduced (Strelow and Brabyn, 1982), but it was demonstrated that although the CB outperformed their sighted blindfolded counterparts, their ability was way below that of the sighted using vision.

**FIGURE 6:**
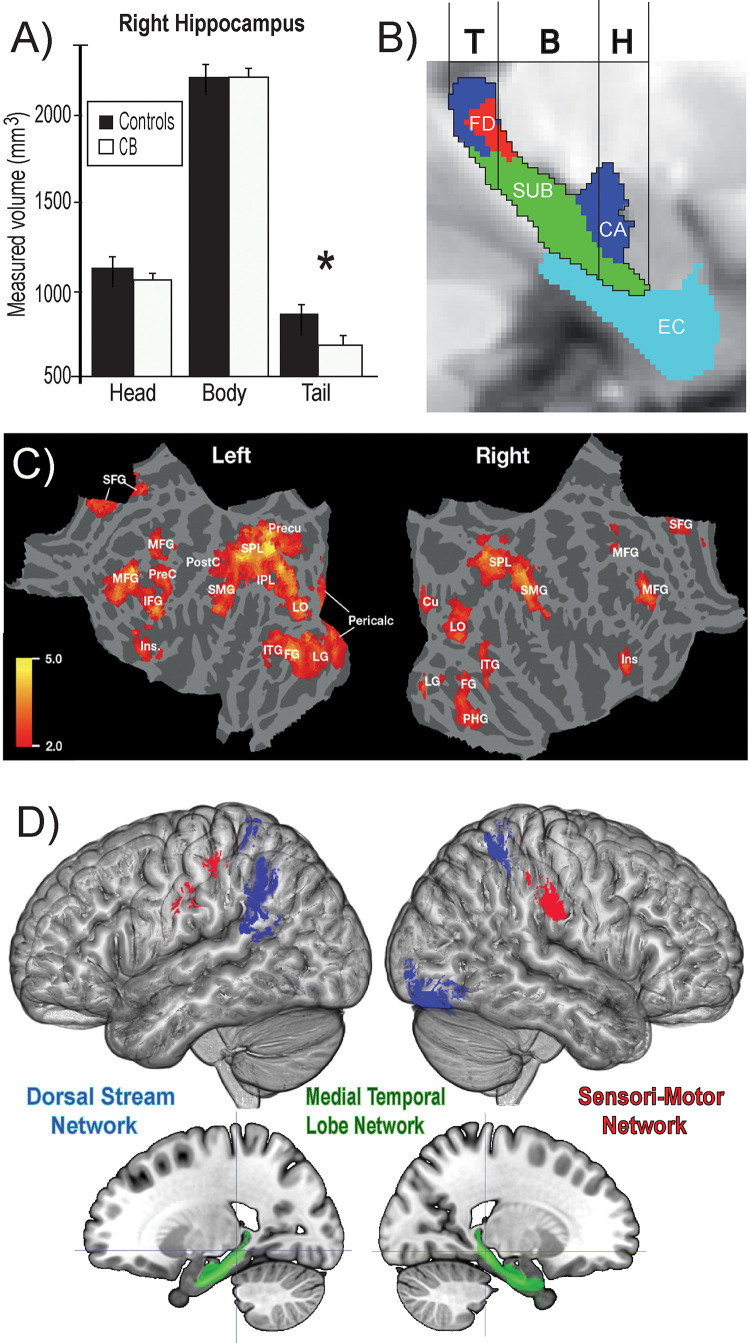
Summary of findings on the neural correlates of navigation in the blind. **(A)** Volumetric reductions in the head of the hippocampus of CB. **(B)** Different cellular layers of the hippocampus according to the head, body and tail segmentation. **(C)** Flat mounts showing recruitment of visual areas for navigation by CB. **(D)** Three networks involved in obstacle detection and avoidance in CB and sighted participants. For avoidance, both CB and SC rely on the dorsal stream network, whereas for obstacle detection SC recruit medial temporal lobe structures and CBs additionally recruit a motor network (adapted from Chebat et al., 2007b, 2020; Kupers et al., 2010a). *p < 0.05.

Theories on the acquisition of spatial competence in blindness can be classified into three main categories, that is either cumulative, persistent or convergent (Figure 5; Schinazi et al., 2016). It is evident that without visual cues, the acquisition of spatial knowledge concerning an environment and eventual spatial competence can be impaired, but to what extent? The cumulative model and persistent models hold that errors made when acquiring spatial knowledge, and thus also spatial competence, in an environment leads to further errors that are either persistently or cumulatively further away from the performance of their sighted counterparts having received as much spatial experience in the same environment. The convergent model considers that although it may take more time for CB people to gain spatial information and spatial competence, eventually their spatial competence will converge with that of the sighted. For a long time, the literature on the subject of congenital blindness has entertained the idea that people who are blind from birth were deficient or ineffective in their ability to comprehend space (von Senden, 1932). The deficiency theory proposes (see both the cumulative and deficient model in Figure 5) that people who are congenitally blind are either incapable of, or inefficient in their ability to develop mental representations of space and environment. According to this theory, this inability to form efficient cognitive maps is due to the use of tactile, proprioceptive, or auditory cues that are not useful in creating these maps. Blindness leads to a diminution in autonomy because of a deficit in orientation in space and mobility. It is evident that it is harder to navigate without the appropriate information furnished by vision. This inability to navigate alone is of course a handicap that is important for blind people (Loomis et al., 1993), who have difficulty in understanding certain concepts relating to space (Rieser et al., 1980), and in making mental rotations (Ungar et al., 1995; Fortin et al., 2006). Further evidence that would seem to support this view comes from volumetric studies of the hippocampus in CB. The posterior end of the right hippocampus is volumetrically reduced in CB (Chebat et al., 2007a; Figure 6A), precisely in the same area that is usually associated with navigation in humans (Duarte et al., 2014). The hippocampus is composed of many different distinct cellular layers (Figure 6B), and it is unknown which ones drive the volumetric reductions in CB. Despite these behavioral findings and volumetric differences, people who are blind, even those without any visual experience, are able to represent familiar spaces, and have an overall good understanding of large spaces (Casey, 1978). In opposition to the *deficiency theories* concerning spatial competence acquisition, there are also many different studies that seem to support the *convergent* model of spatial acquisition. For example, CB process spectral cues more efficiently than the sighted (Doucet et al., 2005), and can process auditory syllables more efficiently (Topalidis et al., 2020), have better sound pitch discrimination (Gougoux et al., 2004), are better at locating sound sources than the sighted (Lessard et al., 1998), more accurate sound localization than the sighted (Lewald, 2007), improved auditory spatial tuning (Röder et al., 1999), and even supra normal auditory abilities in far space (Voss et al., 2004), possibly by recruiting mechanisms of cross-modal brain plasticity to process auditory information (Collignon et al., 2009). Furthermore, it is possible to form a mental layout of space in a virtual task using echo-acoustic information (Dodsworth et al., 2020). It is therefore not a question of a deficit at the level of the mental representation of space. In an environment that does not enable the advantages of visual navigation (i.e., in a maze where the walls were at arm’s length, so that subjects could touch them), the performance of blind subjects was equal to, or even surpassed that of the sighted (Passini et al., 1990; Fortin et al., 2008). Far from being deficient in spatial tasks, nor in their comprehension of space in general, people who are blind may have a different comprehension of space generated by other senses and therefore develop other strategies to represent and configure space (Thinus-Blanc and Gaunet, 1997).

### Spatial Perception Strategies and Sensory Substitution Devices

In the same way that the physiology of the brain shapes vision, the engineering of each different SSD sets limitations on the type and quality of visual information available. The angle of the camera-sensor (field of view) for example or nature of the sensor information (distance information vs. contrast information or edges of objects) and the way this information is conveyed, influence how the SSD user explores the environment (Bermejo et al., 2015). Regardless of the type of visual information transferred, or the modality used by the device (tactile or auditory), the distal attribution process is a crucial step in developing strategies when using SSDs (Siegle and Warren, 2010). This process allows the user to attribute an external cause to the sensation provided by the SSD (Hartcher-O’Brien and Auvray, 2014). When this process is complete, the user is able to understand how the information conveyed by the apparatus relates to the representation of the object in space. This leads to the integration and transformation of SSD information into a coherent representation of the world around us (Cecchetti et al., 2016a) allowing blind people to interact with their environment efficiently. Using the vOICe. for example it is possible to recognize and locate objects efficiently (Brown et al., 2011). The strategies developed by blind people when using SSDs to navigate reflects the absence of a cognitive deficiency in representing space (Schinazi et al., 2016). When vision is substituted by tactile or auditory information the type of strategies used by CB and LB resembles the strategies described above used by the sighted, and the spatial updating of auditory scenes mimics the spatial updating of visual scenes (Pasqualotto and Esenkaya, 2016). Indeed, when comparing the strategies used and navigation patterns of sighted and blindfolded sighted participants using the EyeCane in a virtual environment, we find that LB and CB performances can be quite similar to the sighted. The same is true for the paths they use to explore their environments, using a visual strategy to explore real life Hebb-Williams mazes (Maidenbaum et al., 2014b; Chebat et al., 2015). This is surprising given that much of the spatial information is lost when translated into tactile or auditory information (Richardson et al., 2019). It would seem then that even a little spatial information is enough to enable blind people to develop navigation strategies that resemble those employed by the sighted.

### Perceptions of Obstacles by Congenitally Blind Individuals

Obstacle avoidance tasks include two separate skills. The ability to understand where the obstacle is in space, and also the ability to walk around it. Pointing tasks have for objective the evaluation of knowledge of participants on directional relations between places. These tasks can help to evaluate the perception of space (Kelly et al., 2004), the perception of movement (Israël et al., 1996; Philbeck et al., 2006) and the spatial memory to plan and accomplish a movement. One can ask the participant to move actively or passively and point toward the starting point. We can also ask the subject to verbally describe the azimuth toward the goal. Physically pointing implies the contribution of the motor network to accomplish the motor action of pointing as well as the spatial task to find your point of origin. Navigation also implies the ability to move in the environment and avoid obstacles on the path. Obstacles can be very large, like the size of a mountain or a building for example, that one must skirt (circle) to get around, or quite small, like a sidewalk that one must step over. In both cases, this implies being able to locate the obstacle on the path and develop a strategy to keep the goal in mind and reach it despite this obstacle.

Using tactile information, CBs are able to detect and avoid obstacles efficiently using a SSD in a real life obstacle course (Chebat et al., 2007a, 2011, 2020). Indeed, CBs have natural adaptive mechanisms to use tactile information *in lieu* of visual information. Using the TDU, for example, CBs outperform their sighted blindfolded counterparts in different tasks including navigation. Work from our laboratory using the TDU in route recognition demonstrated the recruitment of primary visual areas in CB, but not in sighted blindfolded or in LB (Kupers et al., 2010a; Figure 6C). In line with these results, CB participants, LB and blindfolded sighted controls learned to use an SSD to navigate in real-life size mazes. We observed that retinotopic regions, including both dorsal-stream regions (e.g., V6) and primary visual cortex regions (e.g., peripheral V1), were selectively recruited for non-visual navigation after the participants mastered the use of the SSD, demonstrating rapid plasticity for non-visual navigation (Maidenbaum et al., 2018). Moreover, the ability of participants to learn to use the SSD to detect and avoid obstacles was positively correlated with the volumes of a network commonly associated with navigation (Chebat et al., 2020; Figure 6D). For avoidance, both CB and SC rely on the dorsal stream network, whereas for obstacle detection SC recruit medial temporal lobe structures and CBs additionally recruit a motor network. These results suggest that the blind may rely more on *motor memory* to remember the location of obstacles (Chebat et al., 2020). Similar results were reported by Gagnon et al. (2010) in a tactile maze where the performance of CBs was significantly higher than that of the sighted controls.

## Future Perspectives of Sensory Substitution Devices

The major conclusion of studies on the blind using SSDs is that navigation is indeed possible without any visual experience. Spatial competence can be achieved by blind individuals partly due to mechanisms of brain plasticity, and amodality. Visual deprivation from birth leads to anatomical volumetric reductions of all components of the visual system, from the retina to the thalamic primary visual relay (dorsal lateral geniculate nucleus) (Cecchetti et al., 2016b), the visual cortex and extrastriate cortices including the ventral and dorsal streams (Ptito et al., 2008b). These structures have been shown to reorganize and develop ectopic projections with other sensory cortices mostly touch and audition (reviewed in Kupers and Ptito, 2014; Chebat et al., 2018b; Harrar et al., 2018). Indeed, CB trained with SSDs activate their primary visual cortex (Ptito, 2005) in a tactile orientation task, and the dorsal visual and ventral streams for tactile motion (Ptito et al., 2009) and the perception of tactile form (Ptito et al., 2012). In line with these findings, another study found retinotopic like maps in the visual cortex of expert blind echolocators, providing further evidence for the task specific organization of the brain (Norman and Thaler, 2019). It seems therefore that CB can compensate for the loss of vision by using other trained senses to invade and recruit the visual cortices. This means that navigational skills are indeed possible through a rewired network of connections that involves the hippocampal/parahippocampal network (Kupers et al., 2010a; Kupers and Ptito, 2014). Furthermore, the use of SSDs could possibly greatly enhance spatial competence in people who are blind by supplementing missing visual information and allowing for the use of more direct exploration of the environment. This would allow blind people to form allocentric representations of space more quickly and efficiently. Indeed, according to a convergent model of spatial competence in CB, by being able to acquire more spatial information in relatively less time via SSDs, CB may be able achieve spatial competence more rapidly.

We conclude here on the future of SSDs and their efficacy for substituting vision in a natural environment. To date, all studies have focused on laboratory settings (Elli et al., 2014) with carefully controlled environments and have furnished encouraging results. However, as all available SSDs suffer from methodological shortcomings from their technology to their adaptability to the environment, it may take a while before we see their widespread use (Chebat et al., 2018a). Current trends investigating the impact of personality traits on SSD use (Richardson et al., 2020) will surely lead to better, more adaptable and customizable devices. Another important question concerns the ideal age to start training with SSDs. Indeed, the developmental aspect is crucial to SSD studies (Aitken and Bower, 1983; Strelow and Warren, 1985), and training children from a very young age could prove to be very beneficial from a behavioral point of view. Most studies using SSDs to explore mechanisms of brain plasticity do so with the training of people well beyond the critical period. Considering that the human brain is much more plastic before the critical period (Cohen et al., 1999; Sadato et al., 2002), it would be very interesting to investigate what congenitally blind children can achieve using SSDs (Strelow and Warren, 1985; Humphrey et al., 1988) compared to sighted adults (Gori et al., 2016). Future studies should also concentrate on studies in acquired blindness in later age, taking into account the onset and duration of blindness. It would also be interesting to investigate the impact of the sophistication (ease of use of devices) and personalization (adapted to each individual) of task specific SSDs.

In order for SSDs to become widespread there is a need to move experiments from the laboratory setting (Elli et al., 2014; Maidenbaum et al., 2014a) to real environments. Also, it would be useful to take advantage of virtual reality to train people with SSDs (Kupers et al., 2010b; Chebat et al., 2015; Baker et al., 2019; Netzer et al., 2019; Yazzolino et al., 2019; Siu et al., 2020) and explore their ability to transfer spatial knowledge between real and virtual environments (Chebat et al., 2017; Guerreiro et al., 2020). Given that these devices are totally non-invasive compared to other highly invasive techniques like surgical implants (retinal or cortical), efforts should be pursued in developing high quality SSDs that will improve the quality of life of the blind.

## Ethics Statement

Written, informed consent was obtained for the publication of any identifiable data and images.

## Author Contributions

All authors collaborated for many years on the topic of sensory substitution and cross modal plasticity, contributed equally to the conception, and writing of this review.

## Conflict of Interest

The authors declare that the research was conducted in the absence of any commercial or financial relationships that could be construed as a potential conflict of interest.
